# Biomechanical Influence of Cartilage Homeostasis in Health and Disease

**DOI:** 10.1155/2011/979032

**Published:** 2011-09-15

**Authors:** D. L. Bader, D. M. Salter, T. T. Chowdhury

**Affiliations:** ^1^School of Engineering and Materials Science, Queen Mary University of London, London E1 4NS, UK; ^2^Department of Pathology, University of Edinburgh, Edinburgh EH4 2XU, UK

## Abstract

There is an urgent demand for long term solutions to improve osteoarthritis treatments in the ageing population. There are drugs that control the pain but none that stop the progression of the disease in a safe and efficient way. Increased intervention efforts, augmented by early diagnosis and integrated biophysical therapies are therefore needed. Unfortunately, progress has been hampered due to the wide variety of experimental models which examine the effect of mechanical stimuli and inflammatory mediators on signal transduction pathways. Our understanding of the early mechanopathophysiology is poor, particularly the way in which mechanical stimuli influences cell function and regulates matrix synthesis. This makes it difficult to identify reliable targets and design new therapies. In addition, the effect of mechanical loading on matrix turnover is dependent on the nature of the mechanical stimulus. Accumulating evidence suggests that moderate mechanical loading helps to maintain cartilage integrity with a low turnover of matrix constituents. In contrast, nonphysiological mechanical signals are associated with increased cartilage damage and degenerative changes. This review will discuss the pathways regulated by compressive loading regimes and inflammatory signals in animal and *in vitro* 3D models. Identification of the chondroprotective pathways will reveal novel targets for osteoarthritis treatments.

## 1. Introduction

It is well established that mechanical loading regulates the structure and function of musculoskeletal tissues and helps maintain the functional integrity of articular cartilage and joint homeostasis. The onset and progression of osteoarthritis (OA) involves all the tissues of the joint initiated by multiple risk factors. These include joint instability and/or misalignment, obesity, previous knee injury, muscle weakness, age, and genetics. It is clear that joint tissues are sensitive to the magnitude, duration, and nature of the mechanical stimulus. A range of approaches have, therefore, been developed to examine the effect of mechanical loading on cartilage homeostasis and OA disease progression. However, each approach has limitations which make it difficult to evaluate the physiological relevance of the experimental findings. This review article will examine the role of abnormal joint loading in cartilage destruction and compare the findings to the protective effects of physiological loading in animal and *in vitro* models. In addition, we will discuss the intracellular mechanisms which mediate the effects of mechanical loading and explore the potential of using controlled exercise therapy in combination with novel agents as an integrated biophysical approach for OA treatments. 

## 2. Influence of Nonphysiological Mechanical Loading and Cartilage Destruction

### 2.1. Joint Overuse and Excessive Mechanical Loading Is Damaging to the Tissue

Cartilage defects in the knees of young or active individuals remain a problem in orthopaedic practice. The clinical symptoms of OA are joint pain, limitation of range of motion, and joint stiffness. Sports activities involving high intensity and repetitive loads increase the risk of OA and are most often associated with other injuries such as knee ligament tears, meniscal injuries, patellae fractures, and osteochondral lesions [1–3]. Cartilage degeneration can develop from direct traumas, joint instability and misalignment, as a result of altered patterns of load distribution across the joint [4]. 

Overloading (e.g., traumatic or high intensity) induces morphological, molecular, and mechanical changes in cells and matrix which leads to softening, fibrillation, ulceration, and loss of cartilage [5–7]. These molecular and biomechanical changes have been shown to shift the balance of tissue remodelling in favour of catabolic over anabolic activity in animal models. However, studies which measure the effects of mechanical loading on cartilage due to overuse in human joints are few in number. By contrast, there are a plethora of experimental studies which have examined the effect of overloading in animal and 3D models (Table 1). For example, strenuous exercise in a canine model caused by running either 20 or 40 km/day for up to 15 weeks reduced proteoglycan content in the superficial zone of cartilage, increased water content, and decreased the concentration of collagen in the load-bearing region [8, 9]. In rodents, enforced running of mice for 1 km/day, or a sudden increase in exercise at an older age resulted in more severe cartilage lesions than observed in sedentary controls [10, 11]. 

*In vitro* studies have identified a critical stress threshold of 15–20 MPa above which cell death and collagen damage was evident due to a single impact load in bovine cartilage explants [12, 13]. In a separate study, apoptosis occurred at peak stresses as low as 4.5 MPa followed by collagen degradation at 7 to 12 MPa and nitrite accumulation at 20 MPa [14]. However, the source of the tissue tested and nature of the impact load will certainly influence the type and extent of damage [15]. For example, human cartilage was found to be more resistant to damage than bovine tissue following a single impact load of similar magnitude [16]. This may be due to the structural differences between the two tissue types and cartilage thickness or effects of age-accumulated changes observed in samples from older patients. Furthermore, impact damage is inevitably strain rate dependent. Indeed, in a comparative study, low strain rates had no discernable effect on matrix synthesis in bovine cartilage explants, whereas strain rates up to twofold higher decreased the levels of proteoglycans, and hence reduced both compressive and shear stiffness [17]. The damage caused by repeated impact loading was cumulative in nature, initially inducing necrosis, followed by apoptosis and collagen degradation in cartilage explants [18, 19]. In addition, the application of static load, equivalent to a compressive strain of 50%, decreased the synthesis of both collagen type II and proteoglycans in bovine cartilage explants [20–22]. Taken together, these experimental findings demonstrate that if joints are insufficiently loaded, cartilage metabolism shifts in favour of catabolism, essentially leading to tissue atrophy. Long-term injurious mechanical loading, which represent high levels of peak stress and/or strain rates, induces abnormal compositional changes in cartilage and accelerates breakdown of the extracellular matrix. However, the magnitude of loads reported *in vitro* may not replicate the *in vivo *loading environment. This limitation makes it difficult to identify the range of nonphysiological loading modalities that are likely to be encountered in a clinical setting. Accordingly, clinical studies of critical patient populations may provide more appropriate means of evaluating the physiological relevance of mechanical factors on integrated disease pathways and treatments.

### 2.2. Reduced Joint Loading and Disuse Leads to Cartilage Degeneration

Reduced joint loading (e.g., static and immobilisation) leads to atrophy and degeneration of cartilage (Table 1). Indeed, animal studies demonstrate that prolonged joint immobilisation causes cartilage thinning, tissue softening, and reduced proteoglycan content resulting in matrix fibrillation, ulceration, and erosion [23–25]. An inactive lifestyle leads to OA like changes in a hamster model, as characterised by reduced proteoglycan content, fibrillation, pitting, and fissuring [26]. In clinical studies, patients with fractures and partial or complete immobilisation presented significant temporal changes in cartilage morphology, including reduced thickness in the femorotibial joint compared to the patella [27]. In the absence of joint loading, patients with spinal cord injuries showed progressive thinning of knee cartilage in the absence of normal joint loading at a rate which was higher than that observed in OA [28, 29]. However, the loss of proteoglycans following short-term immobilisation in a canine model was largely reversible and remobilisation of the joint led to restoration of matrix [30–32]. This effect may be possible, since the loading condition primarily affects the proteoglycan content and does not irreversibly influence the collagen network. However, if the animal is actively exercised at the time of remobilisation, the integrity of the tissue will be compromised, suggesting that prolonged immobilisation may cause irreversible damage to the tissue [33–35].

## 3. Physiological Mechanical Stimuli and Cartilage Homeostasis

### 3.1. Moderate Mechanical Loading Plays a Role in Normal Tissue Remodelling

Several investigators have used a range of approaches to examine the effect of moderate exercise in maintaining cartilage homeostasis (Table 2). Indeed, there is sound evidence that individuals engaging in regular activity are less prone to incidence of OA, since frequent dynamic loading in the physiological range will increase cartilage thickness and maintain normal cartilage integrity [36, 37]. There is also evidence that exercise therapy in the form of aerobic and strengthening activities reduced pain and disability, enhances GAG content, and protects against cartilage degeneration in subjects with knee OA [4, 38–41]. However, the protective effect of recreational exercise has been reported to be dependent on a number of risk factors including age, body mass index, history of knee injury, smoking, and education [42–44]. Clinical observations suggest that healthy subjects as well as OA patients, in general, can pursue a high level of physical activity, provided that the activity is not painful and does not predispose to trauma [45]. However, there is still insufficient information which provides useful guidelines on optimal exercise regimen, dosage, or length of intervention, particularly in overweight individuals [46]. It is interesting to note that prospective cohort studies which examined the effect of exercise on cartilage properties have reported contradictory results [4, 37]. In some patients, the intervention offered pain relief and improvements in physical functioning, but in middle-aged or elderly persons without OA, recreational exercise neither protects against nor increases risk of the disease [42, 43, 47–49]. It is inevitable that comparison of findings between clinical studies, is problematic, as they often involve differences in both diagnostic criteria and variable exercise regimens. However, it is certain that understanding the associations between risk factors and benefits of physical activity will provide key information that will have important implications for clinical practice. 

In most animal studies, load bearing exercise minimises the development of OA. For example, daily exercise increased proteoglycan content and cartilage thickness in hamster and rodent models [26, 50]. In dogs, moderate exercise augments GAG content particularly in younger animals [51, 52]. In hamsters, early joint loading advances the maturation of matrix proteins, improved the integrity of the collagen network and the tissue resistance against OA in older animals [53–56]. In general, exercise and loading of joints within a physiological range appears to have beneficial effects over normal day to day activities characterised by modest movement. The anabolic changes induced by exercise appear to enhance the load bearing properties of cartilage and may help explain how lifelong physical activity protects the joint from OA during later periods in life.

Several *in vitro* studies have examined the effect of physiological mechanical loading on chondrocyte function and matrix synthesis (Table 3). Indeed, stretching of cells in monolayer cultures and compression of chondrocytes in hydrogels or explants generally leads to anabolic signalling cascades and protective effects. For example, aggrecan and collagen type II gene expression was increased by cyclic pressure-induced strain, hydrostatic pressure or fluid-induced shear stress in chondrocyte monolayers [57–59]. In agarose, dynamic compression at low frequencies increased cell proliferation and proteoglycan synthesis following 2 or 21 days of stimulation [60–63]. In cartilage explants, cyclic compression at frequencies of 0.01 to 1 Hz increased proteoglycan synthesis and gene expression of extracellular matrix constituents, aggrecan, fibronectin and cartilage oligomeric matrix protein (COMP) [20, 22, 64–66]. However, results from *in vitro* studies are variable and appear to be dependent on the duration and type of compression regime employed and whether loading was applied during early or late cultures [60, 67–70]. In addition, direct comparison may prove difficult between findings from 3D biomaterial constructs and explant cultures, since both systems have inherent disadvantages. For example, 3D agarose models do not replicate the physiological loading environment of cartilage and are generally cultured for relatively short time periods (hours to weeks). For explants, it is difficult to separate the contribution of the multiple mechanical and physiochemical changes which influence the intracellular pathways and regulate cell function in a spatial and temporal manner. In contrast, *in vivo* animal models facilitate long-term studies within a physiologically relevant environment. However, such models are limited in terms of translating the findings to humans. Nevertheless, information regarding the importance of defining the optimal mechanical parameters required for mechanical conditioning and biosynthesis of anabolic proteins still needs to be established.

## 4. Nonphysiological Mechanical Stimuli and Cartilage Destruction

### 4.1. Abnormal Mechanical Loading Effects on Signal Transduction Pathways

Chondrocytes will respond to excessive mechanical signals by disrupting the composition and structure of the extracellular matrix which reduces the biomechanical integrity of cartilage. Previous *in vitro* studies have demonstrated that mechanical loading, representing an injurious or traumatic response, activates the integrin receptors which stimulate stress-induced intracellular pathways, leading to the production of proinflammatory cytokines such as interleukin-1 (IL-1) and tumour necrosis factor-*α* (TNF*α*). These cytokines disturb the normal remodelling activities of chondrocytes by increasing production of proteolytic enzymes such as matrix metalloproteinases (MMPs) and aggrecanases (ADAMTS), a process mediated by nitric oxide (NO), prostaglandin E_2_ (PGE_2_) and reactive oxygen species (ROS) [71–74]. Furthermore, the enhanced levels of proteinase enzymes cleave both collagens and proteoglycans, resulting in an increase in matrix fragments which stimulate abnormal integrin signals. The accumulation of matrix fragments enhance catabolic protease-driven pathways that override anabolic events and contribute to eventual loss of matrix components and structural damage [75–79]. Abnormal mechanical stimuli are likely to contribute to matrix damage which might shift balance of cell metabolism and lead to the onset of OA. In a recent study, surgical joint destabilisation in rodents for four weeks resulted in an increased expression of TGF*β*2, insulin like growth factor-binding protein (IGF-BP), MMP-2, 12, 13 and 14, ADAMTS5, Toll-like receptor 2 (TLR-2), prostaglandin E synthase (PGES), tumour necrosis factor-stimulated gene 6 (TSG-6), and Wnt-16 [80]. The animal model represents early OA tissue and is in agreement with microarray studies, which revealed changes in MMP-13, COL2A1, and ADAMTS5 in OA cartilage [81, 82]. In addition, several surgical, transgenic or knockout mouse models have provided rapid insights into the mechanisms that control disease progression [83]. In mice, ADAMTS4/5 double knockout and reduced discoidin domain receptor 2 (DDR-2) activation prevents OA progression [84–86]. However, these studies did not provide any information on the mechanical load-induced effects making it difficult to correlate the findings with human disease. 

Recent studies utilised a dietary model of obesity to examine the combined effect of mechanical overload and inflammatory mediators in cartilage degeneration [87, 88]. C57BL/6J mice fed with a high-fat diet increased serum levels of leptin, adiponectin and IL-1*α* leading to degenerative changes observed in knee OA [89]. The studies in mice correlate with clinical findings which found elevated levels of leptin, adiponectin, and resistin in osteoarthritic synovial fluid [90, 91]. The adipokine levels were found to correlate with body mass index and were greater in female subjects, indicating a high prevalence of OA in women [91, 92]. *In vitro* studies demonstrate dose-dependent catabolic and anabolic effects of leptin in chondrocytes, leading to matrix remodelling and/or breakdown. Leptin synergises with IL-1 and was shown to increase NO production leading to MMP activation, apoptosis, and matrix degradation [93, 94]. Furthermore, leptin activates the RhoA/ROCK pathway leading to LIMK1 and cofilin-2 phosphorylation and cytoskeletal re-organisation in chondrocytes [95]. Taken together, these findings indicate that both obesity and mechanical overload influences normal chondrocyte function and contributes to an increase in the incidence and rate of progression of OA.

A number of *in vitro* models have been developed, which examine the effects of various compression regimens on signal transduction pathways. In cartilage explants, 50% static compression for 24 hours increased expression of MMP-3, 9, and 13 and reduced aggrecan and collagen type II within 1 to 2 hours following loading [96–99]. The downregulation of matrix components by static compression was mediated by the interleukin-1 (IL-1) receptor and involves activation of members of the mitogen activation protein kinase (MAPK) family. Static compression differentially stimulates activation of extracellular signal-regulated kinase 1/2 (ERK 1/2), p38 MAPK and SAPK/ERK kinase-1 (SEK1) in a time-dependent manner [96, 100–103]. Several studies demonstrate that IL-1 will additionally stimulate the MAPK pathway and increase MMP levels which reduce proteoglycan synthesis in chondrocytes [104–106]. Both IL-1 and static compression may, therefore, share a global pathway and elicit a catabolic response mediated by members of the MAPK family. However, MAPK activation by static compression was most often transient and was reported to be dependent on the magnitude of applied stress and duration of load [100]. In contrast, cyclic compression for 30 min activates ERK 1/2 and JNK, increased AP-1 binding and expression of MMP-3 and 13, leading to an increase in matrix components in chondrocytes cultured on a calcium polyphosphate substrate [107]. The sequential activation of the MAPK, AP-1, and MMP pathway occurred before matrix degradation and suggests that short-term continuous compression may induce tissue remodelling via these signalling mediators. 

**Figure 1:**
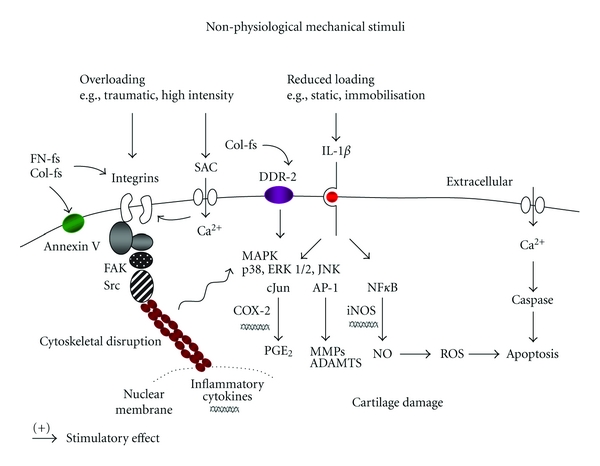
Effect of nonphysiological mechanical stimuli on signal transduction pathways in chondrocytes. Overloading activates the *α*5*β*1 integrin which disrupts the actin cytoskeletal network and stimulates members of the nuclear factor-kappa B (NF*κ*B) and mitogen activated protein kinase (MAPK) family. These factors increase the production of nitric oxide (NO), proteolytic enzymes (MMP-1, 3, 8, and 13), ADAMTS (4 and 5), reactive oxygen species (ROS), cytokines (IL-1, TNF*α*), and prostaglandins (PGE_2_), which mediate cartilage damage. Mechanical signals may indirectly interact with the stretch-activated ion channels (SACs) or increase intracellular calcium levels, which stimulate caspase production (3 and 9) leading to apoptosis. The protease enzymes increase catabolic activities and accelerate tissue damage via production of fibronectin (FN-fs) or collagen fragments (Col-fs), which bind to the integrins, annexin V, discoidin domain receptor 2 (DDR-2), and induce cytokines. Reduced loading (e.g., static and immobilisation) stimulates the IL-1 receptor which activates ERK1/2, AP-1 and MMPs leading to reduced aggrecan and collagen type II synthesis.

The pathways of interactions between non-physiological mechanical signals and inflammatory cytokines will, therefore, involve a number of signalling routes (Figure 1). The actin cytoskeleton plays an important role in mediating the effects of mechanical stimuli on nuclear deformation and cell metabolism. Remodelling of the actin cytoskeleton and disruption of the focal adhesion network leads to focal adhesion kinase (FAK) and Src activation, which stimulate the MAPK cascade. Disruption of the golgi apparatus and cytoskeletal proteins by static compression results in overall loss of mechanical properties leading to a reduction in aggrecan and collagen type II gene expression [108–114]. Tensile or compressive loading at high magnitudes (10 to 15%) for longer periods increased expression of MMP-1, 3, 9, IL-1*β*, and TNF*α* and production of NO and PGE_2_ [115–117]. Upregulation of the inducible nitric oxide synthase (iNOS) and cyclo-oxygenase-2 (COX-2) enzymes will lead to several effects in chondrocytes including increased cytokine production, MMP activation, ROS production, and apoptosis [118–121]. The induction of cell death by fluid-induced shear stress involves protein kinase (PKB) activation and suppression of phosphatidylinositol 3-kinase (PI3-K), which inhibits antioxidant capacity leading to apoptosis [122, 123]. In addition, exposure of T/C-28a2 chondrocytes to high levels of shear stress increased PGE_2_ production and IL-6 expression leading to matrix degradation and chondrocyte apoptosis [124, 125]. The induction of IL-6 was time and magnitude dependent and involved cAMP, protein kinase A (PKA), and PI3-K/Akt-dependent NF*κ*B activation. Prolonged application of shear stress for up to 72 hours increased expression of IL-1*β*, COX-2 and L-prostaglandin D synthase (L-PGDS) leading to ROS production and matrix degradation in T/C-28a2 cells [126]. Furthermore, injurious cyclic or impact loading increased fibronectin synthesis, MMP-3 gene expression, collagen damage, and proteoglycan breakdown in cartilage explants [127, 128]. Overall, these studies demonstrate that the expression of proteins involved in matrix remodelling and catabolism dominate over anabolic signalling events in chondrocytes subjected to abnormal mechanical stimuli.

## 5. Chondroprotective Effects of Mechanical Loading

### 5.1. Signalling Pathways Activated by Physiological Mechanical Stimuli

**Figure 2:**
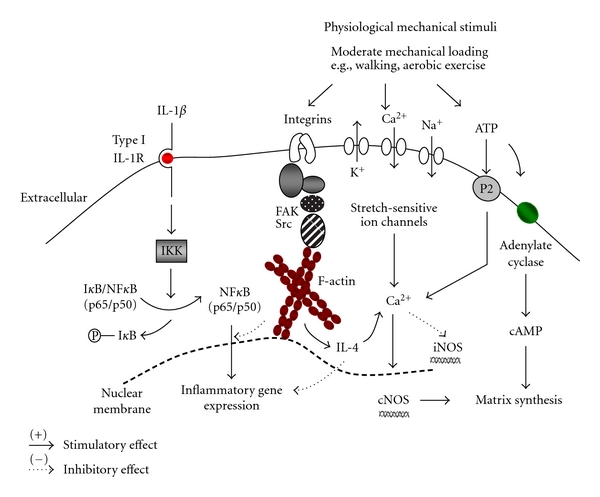
Model depicting the potential protective effects of physiological mechanical stimuli in chondrocytes stimulated with interleukin-1*β* (IL-1*β*). Moderate mechanical loading induces a number of signalling cascades which leads to the production of extracellular matrix components. Mechanical loading will stimulate integrin-mediated release of interleukin-4 (IL-4) via actin cytoskeleton, mechanical perturbation of stretch-sensitive calcium or sodium channels, or stimulation of a purinergic pathway involving ATP release and subsequent purinoreceptor (P2) or cAMP activation. The loading-induced calcium may cause instability of inducible nitric oxide synthase (iNOS) mRNA or increase transport of interleukin-4 (IL-4), which blocks catabolic effects. In the presence of IL-1*β*, mechanical stimuli inhibit cytoplasmic dissociation of NF*κ*B from inhibitory *κ*B-*α* (I*κ*B-*α*), which prevents nuclear translocation of the p65/p50 dimers and/or proteolytic degradation of I*κ*B-*α* by I*κ*B-specific kinases (IKK) or impair I*κ*B-*α* degradation, thereby switching off transcription for the pro-inflammatory genes.

Evidence from *in vitro* studies demonstrate that mechanical signals within a physiological range of intensity, duration and frequency have potent anti-inflammatory effects which counteract the catabolic signals induced by IL-1*β* or TNF*α* (Figure 2). For example, cyclic tensile strain of low magnitudes (3 to 8%) at 0.25 Hz inhibits the expression of iNOS, COX-2, MMP-9 and 13 and increased TIMP-II synthesis in chondrocyte monolayers cultured with IL-1*β* [129]. The downregulation of the catabolic genes by cyclic tensile strain leads to the reduction of NO and PGE_2_ levels and increased synthesis of GAGs, aggrecan and collagen type II [117, 130, 131]. In chondrocytes cultured in agarose constructs, 15% dynamic compression at 1 Hz counteracts IL-1*β*-induced iNOS and COX-2 expression and production of NO and PGE_2_ [132, 133]. Tensile and compressive loading inhibit the nuclear factor-kappa B (NF*κ*B) signal transduction pathway leading to a suppression of iNOS, COX-2, and MMP gene expression [134, 135]. Mechanical stimuli may inhibit cytoplasmic dissociation of NF*κ*B from inhibitory *κ*B-*α* (I*κ*B-*α*), which prevents nuclear translocation of the p65/p50 dimers and/or proteolytic degradation of I*κ*B-*α* by two I*κ*B-specific kinases (IKK*α* and IKK*β*). This effect switches off transcription for the pro-inflammatory genes. Both IL-1*β* and mechanical stimuli interferes with the NF*κ*B cascade and either aggravates or counteracts the induction of several catabolic genes induced by IL-1*β*. However, a noticeable response in the mechanical loading studies was the partial effect of NF*κ*B inhibitors on downregulating the pro-inflammatory response. The gene expression data, therefore, support NF*κ*B-dependent and independent mechanisms, suggesting cross-talk with other pathways. For instance, a number of overlapping genes are regulated by the NF*κ*B and MAPK signal transduction pathways. However, the precise sequence of events which lead to alterations in NF*κ*B or MAPK activity by physiological mechanical signals have yet to be fully explored. 

The critical mechanosensitive components include the integrins and cytoskeletal proteins (Figure 2). Previous *in vitro* studies have shown a role for the integrins in mediating the compression-induced synthesis of matrix components [136–140]. Perturbation of the cell membrane induces integrin conformational changes which promote binding to adaptor proteins (e.g., talin, vinculin, *α*-actinin, paxillin, and zyxin) and interactions with other membrane receptors such as growth factors and stretch-activated ion channels (SACs). The adaptor proteins form the focal adhesion complex which links the integrins to the contractile microfilament bundles, thereby forming a molecular bridge between the extracellular matrix and the cytoskeleton. An intact cytoskeleton is required for normal mechanotransduction and mediates phosphorylation of FAK, paxillin, and Src leading to MAPK activation or secretion of interleukin-4 (IL-4) [137, 138, 141–143]. Substance P is upstream of IL-4 and may act through the NK1 receptor, thereby inducing IL-4 release. It is plausible that IL-4 released through an integrin-mediated mechanotransduction pathway will accumulate and contribute to a pool of soluble anti-inflammatory mediators which block signals induced by IL-1*β*. For example, compressive loading and/or stimulation with IL-4 counteracts IL-1*β* induced NO and PGE_2_ production, MMP-13 expression and stimulates matrix synthesis [144, 145]. Physiological mechanical signals may, therefore, increase the transport of soluble factors which enhance the chondroprotective effects in cartilage.

Moreover, mechanical stimuli may induce ERK due to the release of basic fibroblast growth factor (FGF-2) or cause cell membrane hyperpolarisation leading to an influx of calcium or sodium ions through putative mechanosensitive ion channels [141, 146, 147]. It is possible that membrane deformation induces colocalisation of ion channels with integrin clusters and cytoskeletal complexes resulting in activation of downstream signalling events. For example, the entry of calcium through mechanosensitive ion channels will influence the activity of the constitutive isoform of NOS (cNOS) and regulate calcium/calmodulin binding, PLC/IP_3_ activation, NO production, and aggrecan gene expression [141, 148, 149]. In contrast, the stability of iNOS mRNA has been reported to be reduced by increased calcium, suggesting a possible anabolic route which blocks IL-1*β*-induced signals [150]. Previous studies demonstrate the involvement of a purinergic pathway in mediating mechanical load-induced ATP release and stimulation of anabolic activities [151, 152]. The enhanced ATP levels increased cyclic AMP by adenylate cyclase leading to changes in gene expression [96]. In microarray studies, the increased levels of BMP-2, inhibin *β*A/activin and prostaglandins suggest a possible protective mechanism [82]. Furthermore, the cAMP-responsive element-binding protein (CBP)/p300-interacting transactivator with ED-rich tail 2 (CITED2) has chondroprotective effects and mediates suppression of MMPs in C28/I12 chondrocytes subjected to moderate shear stress or hydrostatic pressure by competing with the transcription factor, Ets-1 [153, 154]. In rodents, one hour of daily passive motion inhibits MMP-1 expression and upregulates CITED2 [154]. These studies clearly identify a number of routes involved in chondroprotection and are illustrated in Figure 2.

## 6. Future Therapies by Combined Exercise and Chondroprotective Agents

The need for novel pharmacological agents which provide effective long-term pain relief and have disease modifying properties for OA treatments is, as yet, unmet. Direct delivery of drugs such as glucocorticoid and hyaluronic acid formulations into the affected joint, do not retard the disease process and may provide only short-term pain relief [155]. The development of novel drugs such as lipid-based formulations and nanoparticles in combination with controlled exercise therapy may provide an alternative strategy in this challenging area of research. Such therapies may be aimed at blocking the pro-inflammatory and proteolytic pathways, thereby allowing the beneficial effects of targeted anabolic exercise regimens. Experimental studies suggest that such options may be of value. For example, intra-articular injection of the IL-1 receptor antagonist (IL-1Ra) was reported to block IL-1*β* actions and reduce OA disease progression in a canine OA model [156]. In rodents, intra-articular injection of IL-4 decreased NO production and prevents cartilage breakdown, whilst in cartilage explants and agarose, growth factors such as IGF-1 or TGF*β* in combination with mechanical stimuli enhance matrix synthesis [144, 145, 157–159]. Furthermore, intra-articular injection of leptin in the rat knee joint stimulates proteoglycan synthesis via increased production of IGF-1 and TGF*β*1 [92]. The combined effect of exercise therapy in conjunction with these chondroprotective agents is not known and merits further investigation. 

A further option is to develop agents which synergise with physiological mechanical loading or which block the signal transduction pathways activated by abnormal mechanical stimuli. Stimulation of mechanoreceptors releases several soluble mediators in chondrocytes including ROS, prostaglandins, cytokines, growth factors, and neuropeptides. These mediators activate downstream signalling events that regulate gene expression and cell function. For example, anti-inflammatory cytokines (IL-4 and IL-10), growth factors (TGF*β*, IGF-1, and FGF-2) and transcriptional regulators (CITED2) synergise with mechanical stimuli and enhance the production of matrix components [144, 145, 154, 160]. Furthermore, physiological mechanical signals antagonise the effects of catabolic mediators involving pro-inflammatory cytokines (IL-1*β*), transcription factors (NF*κ*B), MAPKs, and enzymes (NOS, COX, and MMPs) [133–135, 161, 162]. Mechanical stimulation will increase tension at the cell surface and activate the integrins which are bound to several matrix proteins. Blocking integrin function with antibodies or small molecules has been shown to decrease oxidative damage and improve neurological function following spinal cord injury [163]. Targeting specific ion channels will allow modification of the cells response since mechanical stimuli differentially activates ion channels in normal and OA chondrocytes. Indeed, as the chondrocyte channelome becomes better defined and the roles for these molecules in regulating mechanotransduction become clearer, an increase in the range of potential therapeutic targets will emerge. In this context, N-methyl-D-aspartic acid receptor (NMDAR) may be a novel therapeutic target for OA. NMDAR appears to be necessary for mechanical signalling events in normal and OA chondrocytes [164, 165]. However, the downstream responses are different. In normal chondrocytes, NMDAR mediates mechanical loading-induced activation of small conductance calcium-dependent sodium (SK) channels. In OA chondrocytes, mechanical stimuli opens tetrodotoxin sensitive sodium channels leading to inflammatory gene expression. NMDAR antagonists which block the pro-inflammatory effect are being developed for the treatment of diseases in the central nervous system and drugs such as Memantine are used clinically. Thus, agents which specifically target the functional distinct subtypes of NMDAR will have preferential effects in peripheral tissues and could block mechanical signal induced catabolic pathways. 

In addition, matrix deformation will cause bending of the primary cilia which stimulates connexin 43 (Cx43) hemichannels leading to ATP release and purinergic receptor activation [166, 167]. Deficiency in either the P2X or P2Y receptors will result in reduced responsiveness to mechanical signals leading to new pathways which are catabolic and, therefore, compromise tissue structural properties [168]. Overall, these studies provide further insights on the critical mediators which could be used to promote mechanotherapy for OA. However, pharmacological intervention strategies which either antagonise or enhance the mechanotransduction process are likely to prove difficult. Normal and OA chondrocytes from diseased joints transmit mechanical signals via the *α*5*β*1 integrin, resulting in markedly different downstream signalling events. For example, mechanical stimulation of normal chondrocytes release the chondroprotective IL-4 in contrast to OA cells which produce IL-1*β* [169]. It is possible that chondrocytes from OA cartilage have been reprogrammed to respond differently to their altered mechanical environment, and it may be necessary to target structural components of the cell such as the actin cytoskeleton [170]. This may allow reversal of biomechanical changes developed during OA disease progression allowing the beneficial effects of moderate exercise to be gained at the tissue level.

## 7. Conclusions

The importance of mechanical loading in maintaining healthy joints and normal tissue remodelling has long been recognised. Previous *in vitro* studies continue to support the hypothesis that moderate mechanical loading is necessary to maintain healthy cartilage. If joints are insufficiently loaded, chondrocyte metabolism shifts in favour of catabolism. Similarly, traumatic or excessive joint loading leads to cartilage degeneration and OA. Emerging evidence suggests that physiological joint loading could be used to counteract the inflammatory pathways and restore anabolic activities. Further investigations into the chondroprotective mechanisms are likely to be highly informative and will reveal novel therapeutic targets for OA treatments.

## Figures and Tables

**Table 1:** Experimental evidence indicating the range of nonphysiological loading modalities in articular cartilage.

Type of load	Regimen	Model system	Major effect	Reference
Strenuous exercise	Running 40 km/day for one year	Beagle dogs	Decreased proteoglycan content in load bearing regions	[9]
Strenuous exercise	Running uphill on a treadmill for 40 weeks and 20 km/day for 15 weeks	Beagle dogs	Reduced GAG content in the superficial zone and reduced cartilage thickness	[8]
Immobilisation	3 weeks	Adult dogs	Reduction in proteoglycan synthesis	[23]
Rigid immobilisation	11 weeks	Canine knee	Decrease in cartilage thickness	[24]
Post ankle fracture model of partial load bearing	7 weeks	20 subjects with ankle fractures	Cartilage atrophy and reduced thickness in patellae and medial tibia	[27]
Joints are unloaded and restricted in movement	24 months	26 subjects with traumatic spinal cord injury	Progressive thinning of cartilage in the patella, medial tibia and decrease stiffness	[28, 29]
Immobilisation and remobilisation	Initial 11 week immobilisation and subsequent 50 week remobilisation period	Canine knee	Immobilisation caused softening of tissue Remobilisation partially restored biomechanical properties	[34]
Single impact load	15–20 MPa, 24 hrs	Bovine cartilage explants	Cell death and collagen damage	[12]
Impact load with variable peak stress	4.5 to 20 MPa, 24 hrs	Bovine cartilage explants	Apoptosis (4.5 MPa), collagen breakdown (7–12 MPa), sGAG (6–13 MPa), and nitrite release (20 MPa)	[14]
High strain rate 0.1 and 1/sec	18 and 24 MPa	Bovine cartilage explants	Reduction in protein biosynthesis and compressive/shear stiffness	[17]
High velocity single impact load	24 hrs	Human and bovine cartilage explants	Matrix inhibition was more pronounced in bovine than human tissue	[13, 15, 16]
Repetitive impact load	5 MPa, 0.3 Hz, 2 hrs	Bovine cartilage explants	Necrosis, apoptosis, followed by collagen and proteoglycan degradation	[19]
Static compression	50%, 24 hours	Bovine cartilage explants	Inhibits proteoglycan synthesis and collagen type II	[20, 21]

**Table 2:** Clinical findings supporting a role for exercise therapy in maintaining cartilage health.

Intervention	Duration	Subjects	Outcome	Reference
Aerobic walking and quadriceps strengthening exercise	18 months	35 subjects without knee OA	Both exercise regimen showed normal distribution of proteoglycans and reduced pain and disability from knee OA	[38]
Supervised exercise	3 times weekly for 4 months	45 subjects who underwent partial medial meniscus resection 3–5 years previously	Improved GAG content and reduced pain and joint symptoms	[4]
Cumulative physical exercise	Low (<6862) or high (>8654) exercise hours	805 subjects	Reduced risk in knee OA	[41]
Recreational walking or jogging	Low versus high levels of activity	1279 subjects, with or without knee OA; middle aged or elderly, BMI below or above median	Subjects with a high BMI had no increase in risk of OA. Overweight, middle aged, and elderly persons neither protects against nor increases risk of OA	[42]
Exercise	Various	11 randomised control trials	Beneficial effect on pain and disability	[47]

**Table 3:** A comparison of animal and *in vitro* studies indicating the positive effect of physiological joint loading in articular cartilage.

Type of load	Regimen	Model system	Major effect	Reference
Running exercise	6 to 12 km/day	Hamster	Increased proteoglycan content	[26]
Running exercise	15 km over 28 days	Rat OA induced by ACLT	Reduced apoptosis and chondral erosions	[50]
Running exercise	Varied age, 15 months exercise	Rabbit	Improved collagen organisation in young and reversed OA in older animals	[54]
Increased loading	Increased loading following 8 weeks of splinting	Rabbit	Increased maturation of tissue and increased collagen content	[55]
Conditioning exercise	Increased workload by 30%	Foals	Reduced cartilage degeneration index	[56]
Running exercise	4 km/day, uphill, 15 weeks	Beagle dogs	Increased proteoglycan content and cartilage thickness	[53]
Cyclic pressure-induced strain	0.3 Hz, 6 hours	Human and monolayer	Increased aggrecan gene expression	[58]
Hydrostatic pressure	5 and 10 MPa at 1 Hz for durations of 4 h per day for 4 days	Human monolayer	Increased aggrecan and collagen type II gene expression	[59]
Dynamic compression	3% at 0.01 to 1 Hz, 43 days	Bovine and agarose	Increased proteoglycan and collagen synthesis	[63]
Dynamic compression	15%, 1 Hz, 48 hours	Bovine and agarose	Increased cell proliferation and proteoglycan synthesis and reduced nitrite release	[60, 61]
Dynamic compression	10% at 1 Hz, 3 × 1 hr on, 1 hr off, 5 days/week for 21 days	Bovine and agarose	Increased equilibrium aggregate modulus, sGAG and collagen synthesis	[62]
Dynamic compression	1 MPa, repeated 2 and 4 sec, 1.5 hour	Bovine and explants	Increased proteoglycan synthesis	[64]
Cyclic compression	1 MPa, 0.5 Hz, 3 days	Bovine and explants	Increased proteoglycan synthesis	[70]

## References

[1] Buckwalter JA, Lane NE (1997). Athletics and osteoarthritis. *American Journal of Sports Medicine*.

[2] Stehling C, Luke A, Stahl R (2010). Meniscal T1rho and T2 measured with 3.0T MRI increases directly after running a marathon. *Skeletal Radiology*.

[3] Spector TD, Hart DJ (1996). Radiological evaluation of osteoarthritis. *Revue du Praticien*.

[4] Roos EM, Dahlberg L (2005). Positive effects of moderate exercise on glycosaminoglycan content in knee cartilage: a four-month, randomized, controlled trial in patients at risk of osteoarthritis. *Arthritis and Rheumatism*.

[5] Radin EL, Ehrlich MG, Chernack R, Abernethy P, Paul IL, Rose RM (1978). Effect of repetitive impulsive loading on the knee joints of rabbits. *Clinical Orthopaedics and Related Research*.

[6] Muehleman C, Bareither D, Huch K, Cole AA, Kuettner KE (1997). Prevalence of degenerative morphological changes in the joints of the lower extremity. *Osteoarthritis and Cartilage*.

[7] Mankin HJ, Buckwalter JA (1996). Restoration of the osteoarthrotic joint. *The Journal of Bone and Joint Surgery*.

[8] Kiviranta I, Tammi M, Jurvelin J, Arokoski J, Saamanen AM, Helminen HJ (1992). Articular cartilage thickness and glycosaminoglycan distribution in the canine knee joint after strenuous running exercise. *Clinical Orthopaedics and Related Research*.

[9] Arokoski J, Kiviranta I, Jurvelin J, Tammi M, Helminen HJ (1993). Long-distance running causes site-dependent decrease of cartilage glycosaminoglycan content in the knee joints of beagle dogs. *Arthritis and Rheumatism*.

[10] Lapvetelainen T, Nevalainen T, Parkkinen JJ (1995). Lifelong moderate running training increases the incidence and severity of osteoarthritis in the knee joint of C57BL mice. *Anatomical Record*.

[11] Arokoski JPA, Jurvelin JS, Väätäinen U, Helminen HJ (2000). Normal and pathological adaptations of articular cartilage to joint loading. *Scandinavian Journal of Medicine and Science in Sports*.

[12] Torzilli PA, Grigiene R, Borrelli J, Helfet DL (1999). Effect of impact load on articular cartilage: cell metabolism and viability, and matrix water content. *Journal of Biomechanical Engineering*.

[13] Jeffrey JE, Gregory DW, Aspden RM (1995). Matrix damage and chondrocyte viability following a single impact load on articular cartilage. *Archives of Biochemistry and Biophysics*.

[14] Loening AM, James IE, Levenston ME (2000). Injurious mechanical compression of bovine articular cartilage induces chondrocyte apoptosis. *Archives of Biochemistry and Biophysics*.

[15] Aspden RM, Jeffrey JE, Burgin LV (2002). Impact loading of articular cartilage. *Osteoarthritis and Cartilage*.

[16] Jeffrey JE, Aspden RM (2006). The biophysical effects of a single impact load on human and bovine articular cartilage. *Proceedings of the Institution of Mechanical Engineers H*.

[17] Kurz B, Jin M, Patwari P, Cheng DM, Lark MW, Grodzinsky AJ (2001). Biosynthetic response and mechanical properties of articular cartilage after injurious compression. *Journal of Orthopaedic Research*.

[18] Chen CT, Burton-Wurster N, Lust G, Bank RA, Tekoppele JM (1999). Compositional and metabolic changes in damaged cartilage are peak- stress, stress-rate, and loading-duration dependent. *Journal of Orthopaedic Research*.

[19] Chen CT, Burton-Wurster N, Borden C, Hueffer K, Bloom SE, Lust G (2001). Chondrocyte necrosis and apoptosis in impact damaged articular cartilage. *Journal of Orthopaedic Research*.

[20] Guilak F, Meyer BC, Ratcliffe A, Mow VC (1994). The effects of matrix compression on proteoglycan metabolism in articular cartilage explants. *Osteoarthritis and Cartilage*.

[21] Sah RLY, Kim YJ, Doong JYH, Grodzinsky AJ, Plaas AHK, Sandy JD (1989). Biosynthetic response of cartilage explants to dynamic compression. *Journal of Orthopaedic Research*.

[22] Kim YJ, Grodzinsky AJ, Plaas AHK (1996). Compression of cartilage results in differential effects on biosynthetic pathways for aggrecan, link protein, and hyaluronan. *Archives of Biochemistry and Biophysics*.

[23] Palmoski M, Perricone E, Brandt KD (1979). Development and reversal of a proteoglycan aggregation defect in normal canine knee cartilage after immobilization. *Arthritis and Rheumatism*.

[24] Jurvelin J, Kiviranta I, Tammi M, Helminen JH (1986). Softening of canine articular cartilage after immobilization of the knee joint. *Clinical Orthopaedics and Related Research*.

[25] Copray JC, Jansen HWB, Duterloo HS (1985). Effects of compressive forces on proliferation and matrix synthesis in mandibular condylar cartilage of the rat in vitro. *Archives of Oral Biology*.

[26] Otterness IG, Eskra JD, Bliven ML, Shay AK, Pelletier JP, Milici AJ (1998). Exercise protects against articular cartilage degeneration in the hamster. *Arthritis and Rheumatism*.

[27] Hinterwimmer S, Krammer M, Krötz M (2004). Cartilage atrophy in the knees of patients after seven weeks of partial load bearing. *Arthritis and Rheumatism*.

[28] Vanwanseele, Eckstein F, Knecht H, Stüssi E, Spaepen A (2002). Knee cartilage of spinal cord-injured patients displays progressive thinning in the absence of normal joint loading and movement. *Arthritis and Rheumatism*.

[29] Vanwanseele B, Eckstein F, Knecht H, Spaepen A, Stüssis E (2003). Longitudinal analysis of cartilage atrophy in the knees of patients with spinal cord injury. *Arthritis and Rheumatism*.

[30] Haapala J, Arokoski JPA, Hyttinen MM (1999). Remobilization does not fully restore immobilization induced articular cartilage atrophy. *Clinical Orthopaedics and Related Research*.

[31] Saamanen AM, Tammi M, Jurvelin J, Kiviranta I, Helminen HJ (1990). Proteoglycan alterations following immobilization and remobilization in the articular cartilage of young canine knee (stifle) joint. *Journal of Orthopaedic Research*.

[32] Jurvelin J, Kiviranta I, Saamanen AM, Tammi M, Helminen HJ (1989). Partial restoration of immobilization-inducing softening of canine articular cartilage after remobilization of the knee (stifle) joint. *Journal of Orthopaedic Research*.

[33] Palmoski MJ, Brandt KD (1981). Running inhibits the reversal of atrophic changes in canine knee cartilage after removal of a leg cast. *Arthritis and Rheumatism*.

[34] Haapala J, Arokoski J, Pirttimäki J (2000). Incomplete restoration of immobilization induced softening of young beagle knee articular cartilage after 50-week remobilization. *International Journal of Sports Medicine*.

[35] Buckwalter JA (1998). Articular cartilage: injuries and potential for healing. *Journal of Orthopaedic and Sports Physical Therapy*.

[36] Jordan KM, Arden NK, Doherty M (2003). EULAR recommendations 2003: rn evidence based approach to the management of knee osteoarthritis: report of a task force of the Standing Committee for International Clinical Studies including therapeutic trials (ESCISIT). *Annals of the Rheumatic Diseases*.

[37] Fransen M, McConnell S, Bell M (2002). Therapeutic exercise for people with osteoarthritis of the hip or knee. A systematic review. *Journal of Rheumatology*.

[38] Roddy E, Zhang W, Doherty M (2005). Aerobic walking or strengthening exercise for osteoarthritis of the knee? A systematic review. *Annals of the Rheumatic Diseases*.

[39] Jansen MJ, Viechtbauer W, Lenssen AF, Hendriks EJ, de Bie RA (2011). Strength training alone, exercise therapy alone, and exercise therapy with passive manual mobilisation each reduce pain and disability in people with knee osteoarthritis: a systematic review. *Journal of Physiotherapy*.

[40] Jansen MJ, Hendriks EJ, Oostendorp RAB, Dekker J, De Bie RA (2010). Quality indicators indicate good adherence to the clinical practice guideline on “Osteoarthritis of the hip and knee” and few prognostic factors influence outcome indicators: a prospective cohort study. *European Journal of Physical and Rehabilitation Medicine*.

[41] Manninen P, Riihimaki H, Heliovaara M, Suomalainen O (2001). Physical exercise and risk of severe knee OA requiring arthriplasty. *Rheumatology*.

[42] Felson DT, Niu J, McClennan C (2007). Knee buckling: prevalence, risk factors, and associated limitations in function. *Annals of Internal Medicine*.

[43] Messier SP (2010). Diet and exercise for obese adults with knee osteoarthritis. *Clinics in Geriatric Medicine*.

[44] Perrot S, Poiraudeau S, Kabir-Ahmadi M, Rannou F (2009). Correlates of pain intensity in Men and Women with hip and knee osteoarthritis. Results of a national survey: the French ARTHRIX study. *Clinical Journal of Pain*.

[45] Vignon E, Valat JP, Rossignol M (2006). Osteoarthritis of the knee and hip and activity: a systematic international review and synthesis (OASIS). *Joint Bone Spine*.

[46] Bennell KL, Hinman RS (2011). A review of the clinical evidence for exercise in osteoarthritis of the hip and knee. *Journal of Science and Medicine in Sport*.

[47] van Baar ME, Assendelft WJJ, Dekker J, Oostendorp RAB, Bijlsma JWJ (1999). Effectiveness of exercise therapy in patients with osteoarthritis of the hip or knee: a systematic review of randomized clinical trials. *Arthritis and Rheumatism*.

[48] Ettinger WH, Burns R, Messier SP (1997). A randomized trial comparing aerobic exercise and resistance exercise with a health education program in older adults with knee osteoarthritis: the Fitness Arthritis and Seniors Trial (FAST). *Journal of the American Medical Association*.

[49] Creaby MW, Wang Y, Bennell KL (2010). Dynamic knee loading is related to cartilage defects and tibial plateau bone area in medial knee osteoarthritis. *Osteoarthritis and Cartilage*.

[50] Galois L, Etienne S, Grossin L (2003). Moderate-impact exercise is associated with decreased severity of experimental osteoarthritis in rats (multiple letters). *Rheumatology*.

[51] Buckwalter JA (1995). Osteoarthritis and articular cartilage use, disuse, and abuse: experimental studies. *Journal of Rheumatology*.

[52] Kiviranta I, Tammi M, Jurvelin J, Saamanen AM, Helminen HJ (1988). Moderate running exercise augments glycosaminoglycans and thickness of articular cartilage in the knee joint of young Beagle dogs. *Journal of Orthopaedic Research*.

[53] Helminen HJ, Hyttinen MM, Lammi MJ (2000). Regular joint loading in youth assists in the establishment and strengthening of the collagen network of articular cartilage and contributes to the prevention of osteoarthrosis later in life: a hypothesis. *Journal of Bone and Mineral Metabolism*.

[54] Julkunen P, Iivarinen J, Brama PA, Arokoski J, Jurvelin JS, Helminen HJ (2010). Maturation of collagen fibril network structure in tibial and femoral cartilage of rabbits. *Osteoarthritis and Cartilage*.

[55] Säämänen AM, Tammi M, Kiviranta I, Jurvelin J, Helminen HJ (1987). Maturation of proteoglycan matrix in articular cartilage under increased and decreased joint loading. A study in young rabbits. *Connective Tissue Research*.

[56] van Weeren PR, Firth EC, Brommer H (2008). Early exercise advances the maturation of glycosaminoglycans and collagen in the extracellular matrix of articular cartilage in the horse. *Equine Veterinary Journal*.

[57] Das P, Schurman DJ, Smith RL (1997). Nitric oxide and G proteins mediate the response of bovine articular chondrocytes to fluid-induced shear. *Journal of Orthopaedic Research*.

[58] Millward-Sadler SJ, Wright MO, Davies LW, Nuki G, Salter DM (2000). Mechanotransduction via integrins and interleukin-4 results in altered aggrecan and matrix metalloproteinase 3 gene expression in normal, but not osteoarthritic, human articular chondrocytes. *Arthritis and Rheumatism*.

[59] Ikenoue T, Trindade MCD, Lee MS (2003). Mechanoregulation of human articular chondrocyte aggrecan and type II collagen expression by intermittent hydrostatic pressure in vitro. *Journal of Orthopaedic Research*.

[60] Lee DA, Bader DL (1997). Compressive strains at physiological frequencies influence the metabolism of chondrocytes seeded in agarose. *Journal of Orthopaedic Research*.

[61] Lee DA, Frean SP, Lees P, Bader DL (1998). Dynamic mechanical compression influences nitric oxide production by articular chondrocytes seeded in agarose. *Biochemical and Biophysical Research Communications*.

[62] Mauck RL, Soltz MA, Wang CCB (2000). Functional tissue engineering of articular cartilage through dynamic loading of chondrocyte-seeded agarose gels. *Journal of Biomechanical Engineering*.

[63] Buschmann MD, Gluzband YA, Grodzinsky AJ, Hunziker EB (1995). Mechanical compression modulates matrix biosynthesis in chondrocyte/agarose culture. *Journal of Cell Science*.

[64] Parkkinen JJ, Lammi MJ, Helminen HJ, Tammi M (1992). Local stimulation of proteoglycan synthesis in articular cartilage explants by dynamic compression in vitro. *Journal of Orthopaedic Research*.

[65] Valhmu WB, Stazzone EJ, Bachrach NM (1998). Load-controlled compression of articular cartilage induces a transient stimulation of aggrecan gene expression. *Archives of Biochemistry and Biophysics*.

[66] Wong M, Siegrist M, Cao X (1999). Cyclic compression of articular cartilage explants is associated with progressive consolidation and altered expression pattern of extracellular matrix proteins. *Matrix Biology*.

[67] Sauerland K, Raiss RX, Steinmeyer J (2003). Proteoglycan metabolism and viability of articular cartilage explants as modulated by the frequency of intermittent loading. *Osteoarthritis and Cartilage*.

[68] Chowdhury TT, Bader DL, Shelton JC, Lee DA (2003). Temporal regulation of chondrocyte metabolism in agarose constructs subjected to dynamic compression. *Archives of Biochemistry and Biophysics*.

[69] Ackermann B, Steinmeyer J (2005). Collagen biosynthesis of mechanically loaded articular cartilage explants. *Osteoarthritis and Cartilage*.

[70] Steinmeyer J, Knue S, Raiss RX, Pelzer I (1999). Effects of intermittently applied cyclic loading on proteoglycan metabolism and swelling behaviour of articular cartilage explants. *Osteoarthritis and Cartilage*.

[71] Fernandes JC, Martel-Pelletier J, Pelletier JP (2002). The role of cytokines in osteoarthritis pathophysiology. *Biorheology*.

[72] Henrotin YE, Bruckner P, Pujol JPL (2003). The role of reactive oxygen species in homeostasis and degradation of cartilage. *Osteoarthritis and Cartilage*.

[73] Kim HA, Blanco FJ (2007). Cell death and apoptosis in ostearthritic cartilage. *Current Drug Targets*.

[74] Martel-Pelletier J, Boileau C, Pelletier JP, Roughley PJ (2008). Cartilage in normal and osteoarthritis conditions. *Best Practice and Research: Clinical Rheumatology*.

[75] Homandberg GA (1997). Potential regulation of cartilage metabolism in osteoarthritis by fibronectin fragments. In Fundamental pathways in osteoarthritis. Edited by Malemud CJ. *Frontiers in Bioscience*.

[76] Xie DL, Hui F, Homandberg GA (1994). Cartilage chondrolysis by fibronectin fragments is associated with release of several proteinases: Stromelysin plays a major role in chondrolysis. *Archives of Biochemistry and Biophysics*.

[77] Del Carlo M, Schwartz D, Erickson EA, Loeser RF (2007). Endogenous production of reactive oxygen species is required for stimulation of human articular chondrocyte matrix metalloproteinase production by fibronectin fragments. *Free Radical Biology and Medicine*.

[78] Stanton H, Ung L, Fosang AJ (2002). The 45 kDa collagen-binding fragment of fibronectin induces matrix metalloproteinase-13 synthesis by chondrocytes and aggrecan degradation by aggrecanases. *Biochemical Journal*.

[79] Guo D, Ding L, Homandberg GA (2009). Telopeptides of type II collagen upregulate proteinases and damage cartilage but are less effective than highly active fibronectin fragments. *Inflammation Research*.

[80] Appleton CTG, Pitelka V, Henry J, Beier F (2007). Global analyses of gene expression in early experimental osteoarthritis. *Arthritis and Rheumatism*.

[81] Dell’Accio F, De Bari C, Eltawil NM, Vanhummelen P, Pitzalis C (2008). Identification of the molecular response of articular cartilage to injury, by microarray screening: Wnt-16 expression and signaling after injury and in osteoarthritis. *Arthritis and Rheumatism*.

[82] Aigner T, Fundel K, Saas J (2006). Large-scale gene expression profiling reveals major pathogenetic pathways of cartilage degeneration in osteoarthritis. *Arthritis and Rheumatism*.

[83] Helminen HJ, Säämänen AM, Salminen H, Hyttinen MM (2002). Transgenic mouse models for studying the role of cartilage macromolecules in osteoarthritis. *Rheumatology*.

[84] Xu L, Peng H, Glasson S (2007). Increased expression of the collagen receptor discoidin domain receptor 2 in articular cartilage as a key event in the pathogenesis of osteoarthritis. *Arthritis and Rheumatism*.

[85] Majumdar MK, Askew R, Schelling S (2007). Double-knockout of ADAMTS-4 and ADAMTS-5 in mice results in physiologically normal animals and prevents the progression of osteoarthritis. *Arthritis and Rheumatism*.

[86] Glasson SS (2007). In vivo osteoarthritis target validation utilizing genetically-modified mice. *Current Drug Targets*.

[87] Griffin TM, Guilak F (2008). Why is obesity associated with osteoarthritis? Insights from mouse models of obesity. *Biorheology*.

[88] Pottie P, Presle N, Terlain B, Netter P, Mainard D, Berenbaum F (2006). Obesity and osteoarthritis: more complex than predicted!. *Annals of the Rheumatic Diseases*.

[89] Griffin TM, Fermor B, Huebner JL (2010). Diet-induced obesity differentially regulates behavioral, biomechanical, and molecular risk factors for osteoarthritis in mice. *Arthritis Research and Therapy*.

[90] Schäffler A, Ehling A, Neumann E (2003). Adipocytokines in synovial fluid. *Journal of the American Medical Association*.

[91] Presle N, Pottie P, Dumond H (2006). Differential distribution of adipokines between serum and synovial fluid in patients with osteoarthritis. Contribution of joint tissues to their articular production. *Osteoarthritis and Cartilage*.

[92] Dumond H, Presle N, Terlain B (2003). Evidence for a key role of leptin in osteoarthritis. *Arthritis and Rheumatism*.

[93] Otero M, Lago R, Lago F, Reino JJ, Gualillo O (2005). Signalling pathway involved in nitric oxide synthase type II activation in chondrocytes: synergistic effect of leptin with interleukin-1. *Arthritis Research and Therapy*.

[94] Koskinen A, Vuolteenaho K, Nieminen R, Moilanen T, Moilanen E (2011). Leptin enhances MMP-1, MMP-3 and MMP-13 production in human osteoarthritic cartilage and correlates with MMP-1 and MMP-3 in synovial fluid from OA patients. *Clinical and Experimental Rheumatology*.

[95] Liang J, Feng J, Wu WK (2011). Leptin-mediated cytoskeletal remodeling in chondrocytes occurs via the RhoA/ROCK pathway. *Journal of Orthopaedic Research*.

[96] Fitzgerald JB, Jin M, Dean D, Wood DJ, Zheng MH, Grodzinsky AJ (2004). Mechanical compression of cartilage explants induces multiple time-dependent gene expression patterns and involves intracellular calcium and cyclic AMP. *Journal of Biological Chemistry*.

[97] Murata M, Bonassar LJ, Wright M, Mankin HJ, Towle CA (2003). A role for the interleukin-1 receptor in the pathway linking static mechanical compression to decreased proteoglycan synthesis in surface articular cartilage. *Archives of Biochemistry and Biophysics*.

[98] Fehrenbacher A, Steck E, Rickert M, Roth W, Richter W (2003). Rapid regulation of collagen but not metalloproteinase 1, 3, 13, 14 and tissue inhibitor of metalloproteinase 1, 2, 3 expression in response to mechanical loading of cartilage explants in vitro. *Archives of Biochemistry and Biophysics*.

[99] Ragan PM, Badger AM, Cook M (1999). Down-regulation of chondrocyte aggrecan and type-II collagen gene expression correlates with increases in static compression magnitude and duration. *Journal of Orthopaedic Research*.

[100] Fanning PJ, Emkey G, Smith RJ, Grodzinsky AJ, Szasz N, Trippel SB (2003). Mechanical regulation of mitogen-activated protein kinase signaling in articular cartilage. *Journal of Biological Chemistry*.

[101] Li KW, Wang AS, Sah RL (2003). Microenvironment regulation of extracellular signal-regulated kinase activity in chondrocytes: effects of culture configuration, interleukin-1, and compressive stress. *Arthritis and Rheumatism*.

[102] Bougault C, Paumier A, Aubert-Foucher E, Mallein-Gerin F (2008). Molecular analysis of chondrocytes cultured in agarose in response to dynamic compression. *BMC Biotechnology*.

[103] Ding L, Heying E, Nicholson N (2010). Mechanical impact induces cartilage degradation via mitogen activated protein kinases. *Osteoarthritis and Cartilage*.

[104] Mengshol JA, Vincenti MP, Coon CI, Barchowsky A, Brinckerhoff CE (2000). Interleukin-1 induction of collagenase 3 (matrix metalloproteinase 13) gene expression in chondrocytes requires p38, c-Jun N-terminal kinase, and nuclear factor kappaB: differential regulation of collagenase 1 and collagenase 3. *Arthritis and Rheumatism*.

[105] Liacini A, Sylvester J, Li WQ (2003). Induction of matrix metalloproteinase-13 gene expression by TNF-*α* is mediated by MAP kinases, AP-1, and NF-*κ*B transcription factors in articular chondrocytes. *Experimental Cell Research*.

[106] Goldring MB, Krane SM (1987). Modulation by recombinant interleukin 1 of synthesis of types I and III collagens and associated procollagen mRNA levels in cultured human cells. *Journal of Biological Chemistry*.

[107] De Croos JNA, Dhaliwal SS, Grynpas MD, Pilliar RM, Kandel RA (2006). Cyclic compressive mechanical stimulation induces sequential catabolic and anabolic gene changes in chondrocytes resulting in increased extracellular matrix accumulation. *Matrix Biology*.

[108] Jortikka MO, Parkkinen JJ, Inkinen RI (2000). The role of microtubules in the regulation of proteoglycan synthesis in chondrocytes under hydrostatic pressure. *Archives of Biochemistry and Biophysics*.

[109] Parkkinen JJ, Lammi MJ, Pelttari A, Helminen HJ, Tammi M, Virtanen I (1993). Altered Golgi apparatus in hydrostatically loaded articular cartilage chondrocytes. *Annals of the Rheumatic Diseases*.

[110] Blain EJ, Gilbert SJ, Hayes AJ, Duance VC (2006). Disassembly of the vimentin cytoskeleton disrupts articular cartilage chondrocyte homeostasis. *Matrix Biology*.

[111] Idowu BD, Knight MM, Bader DL, Lee DA (2000). Confocal analysis of cytoskeletal organisation within isolated chondrocyte sub-populations cultured in agarose. *Histochemical Journal*.

[112] Lee DA, Knight MM, Bolton JF, Idowu BD, Kayser MV, Bader DL (2000). Chondrocyte deformation within compressed agarose constructs at the cellular and sub-cellular levels. *Journal of Biomechanics*.

[113] Haudenschild DR, Chen J, Pang N (2011). Vimentin contributes to changes in chondrocyte stiffness in osteoarthritis. *Journal of Orthopaedic Research*.

[114] Trickey WR, Vail TP, Guilak F (2004). The role of the cytoskeleton in the viscoelastic properties of human articular chondrocytes. *Journal of Orthopaedic Research*.

[115] Honda K, Ohno S, Tanimoto K (2000). The effects of high magnitude cyclic tensile load on cartilage matrix metabolism in cultured chondrocytes. *European Journal of Cell Biology*.

[116] Fermor B, Brice Weinberg J, Pisetsky DS, Misukonis MA, Banes AJ, Guilak F (2001). The effects of static and intermittent compression on nitric oxide production in articular cartilage explants. *Journal of Orthopaedic Research*.

[117] Long P, Gassner R, Agarwal S (2001). Tumor necrosis factor alpha-dependent proinflammatory gene induction is inhibited by cyclic tensile strain in articular chondrocytes in vitro. *Arthritis and Rheumatism*.

[118] Lotz M, Hashimoto S, Kühn K (1999). Mechanisms of chondrocyte apoptosis. *Osteoarthritis and Cartilage*.

[119] Studer R, Jaffurs D, Stefanovic-Racic M, Robbins PD, Evans CH (1999). Nitric oxide in osteoarthritis. *Osteoarthritis and Cartilage*.

[120] Martel-Pelletier J, Alaaeddine N, Pelletier JP (1999). Cytokines and their role in the pathophysiology of osteoarthritis. *Frontiers in Bioscience*.

[121] Henrotin Y, Kurz B, Aigner T (2005). Oxygen and reactive oxygen species in cartilage degradation: friends or foes?. *Osteoarthritis and Cartilage*.

[122] Healy ZR, Lee NH, Gao X (2005). Divergent responses of chondrocytes and endothelial cells to shear stress: cross-talk among COX-2, the phase 2 response, and apoptosis. *Proceedings of the National Academy of Sciences of the United States of America*.

[123] Borrelli J, Tinsley K, Ricci WM, Burns M, Karl IE, Hotchkiss R (2003). Induction of chondrocyte apoptosis following impact load. *Journal of Orthopaedic Trauma*.

[124] Wang P, Zhu F, Lee NH, Konstantopoulos K (2010). Shear-induced interleukin-6 synthesis in chondrocytes: roles of E prostanoid (EP) 2 and EP3 in cAMP/protein kinase A- and PI3-K/Akt-dependent NF-*κ*B activation. *Journal of Biological Chemistry*.

[125] Wang P, Zhu F, Konstantopoulos K (2010). Prostaglandin E2 induces interleukin-6 expression in human chondrocytes via cAMP/protein kinase A- and phosphatidylinositol 3-kinase-dependent NF-*κ*B activation. *American Journal of Physiology*.

[126] Zhu F, Wang P, Lee NH, Goldring MB, Konstantopoulos K (2010). Prolonged application of high fluid shear to chondrocytes recapitulates gene expression profiles associated with osteoarthritis. *PLoS ONE*.

[127] Lin PM, Chen CTC, Torzilli PA (2004). Increased stromelysin-1 (MMP-3), proteoglycan degradation (3B3- and 7D4) and collagen damage in cyclically load-injured articular cartilage. *Osteoarthritis and Cartilage*.

[128] Steinmeyer J, Ackermann B (1999). The effect of continuously applied cyclic mechanical loading on the fibronectin metabolism of articular cartilage explants. *Research in Experimental Medicine*.

[129] Madhavan S, Anghelina M, Rath-Deschner B (2006). Biomechanical signals exert sustained attenuation of proinflammatory gene induction in articular chondrocytes. *Osteoarthritis and Cartilage*.

[130] Gassner R, Buckley MJ, Georgescu H (1999). Cyclic tensile stress exerts anti-inflammatory actions on chondrocytes by inhibiting inducible nitric oxide synthase. *Journal of Immunology*.

[131] Xu Z, Buckley MJ, Evans CH, Agarwal S (2000). Cyclic tensile strain acts as an antagonist of IL-1 beta actions in chondrocytes. *Journal of Immunology*.

[132] Chowdhury TT, Bader DL, Lee DA (2001). Dynamic compression inhibits the synthesis of nitric oxide and PGE2 by IL-1*β* stimulated chondrocytes cultured in agarose constructs. *Biochemical and Biophysical Research Communications*.

[133] Chowdhury TT, Arghandawi S, Brand J (2008). Dynamic compression counteracts IL-1*β* induced inducible nitric oxide synthase and cyclo-oxygenase-2 expression in chondrocyte/agarose constructs. *Arthritis Research and Therapy*.

[134] Akanji OO, Sakthithasan P, Salter DM, Chowdhury TT (2010). Dynamic compression alters NFkappaB activation and IkappaB-alpha expression in IL-1beta-stimulated chondrocyte/agarose constructs. *Inflammation Research*.

[135] Agarwal S, Deschner J, Long P (2004). Role of NF-kappaB transcription factors in anti-inflammatory and pro-inflammatory actions of mechanical signals. *Arthritis and Rheumatism*.

[136] Millward-Sadler SJ, Wright MO, Lee HS (1999). Integrin-regulated secretion of interleukin 4: a novel pathway of mechanotransduction in human articular chondrocytes. *Journal of Cell Biology*.

[137] Millward-Sadler SJ, Khan NS, Bracher MG, Wright MO, Salter DM (2006). Roles for the interleukin-4 receptor and associated JAK/STAT proteins in human articular chondrocyte mechanotransduction. *Osteoarthritis and Cartilage*.

[138] Salter DM, Millward-Sadler SJ, Nuki G, Wright MO (2001). Integrin-interleukin-4 mechanotransduction pathways in human chondrocytes. *Clinical Orthopaedics and Related Research*.

[139] Chowdhury TT, Salter DM, Bader DL, Lee DA (2004). Integrin-mediated mechanotransduction processes in TGF*β*-stimulated monolayer-expanded chondrocytes. *Biochemical and Biophysical Research Communications*.

[140] Chowdhury TT, Appleby RN, Salter DM, Bader DA, Lee DA (2006). Integrin-mediated mechanotransduction in IL-1*β* stimulated chondrocytes. *Biomechanics and Modeling in Mechanobiology*.

[141] Wright MO, Nishida K, Bavington C (1997). Hyperpolarisation of cultured human chondrocytes following cyclical pressure-induced strain: evidence of a role for *α*5*β*1 integrin as a chondrocyte mechanoreceptor. *Journal of Orthopaedic Research*.

[142] Lee HS, Millward-Sadler SJ, Wright MO, Nuki G, Salter DM (2000). Integrin and mechanosensitive ion channel-dependent tyrosine phosphorylation of focal adhesion proteins and *β*-catenin in human articular chondrocytes after mechanical stimulation. *Journal of Bone and Mineral Research*.

[143] Knight MM, Toyoda T, Lee DA, Bader DL (2006). Mechanical compression and hydrostatic pressure induce reversible changes in actin cytoskeletal organisation in chondrocytes in agarose. *Journal of Biomechanics*.

[144] Chowdhury TT, Bader DL, Lee DA (2006). Anti-inflammatory effects of IL-4 and dynamic compression in IL-1*β* stimulated chondrocytes. *Biochemical and Biophysical Research Communications*.

[145] Doi H, Nishida K, Yorimitsu M (2008). Interleukin-4 downregulates the cyclic tensile stress-induced matrix metalloproteinases-13 and cathepsin b expression by rat normal chondrocytes. *Acta Medica Okayama*.

[146] Vincent T, Hermansson M, Bolton M, Wait R, Saklatvala J (2002). Basic FGF mediates an immediate response of articular cartilage to mechanical injury. *Proceedings of the National Academy of Sciences of the United States of America*.

[147] Vincent TL, McLean CJ, Full LE, Peston D, Saklatvala J (2007). FGF-2 is bound to perlecan in the pericellular matrix of articular cartilage, where it acts as a chondrocyte mechanotransducer. *Osteoarthritis and Cartilage*.

[148] Wright MO, Nishida K, Bavington C (1997). Hyperpolarisation of cultured human chondrocytes following cyclical pressure-induced strain: evidence of a role for alpha 5 beta 1 integrin as a chondrocyte mechanoreceptor. *Journal of Orthopaedic Research*.

[149] Valhmu WB, Raia FJ (2002). myo-inositol 1,4,5-trisphosphate and Ca2+/calmodulin-dependent factors mediate transduction of compression-induced signals in bovine articular chondrocytes. *Biochemical Journal*.

[150] Geng Y, Lotz M (1995). Increased intracellular Ca2+ selectively suppresses IL-1-induced NO production by reducing iNOS mRNA stability. *Journal of Cell Biology*.

[151] Millward-Sadler SJ, Wright MO, Flatman PW, Salter DM (2004). ATP in the mechanotransduction pathway of normal human chondrocytes. *Biorheology*.

[152] Chowdhury TT, Knight MM (2006). Purinergic pathway suppresses the release of NO and stimulates proteoglycan synthesis in chondrocyte/agarose constructs subjected to dynamic compression. *Journal of Cellular Physiology*.

[153] Yokota H, Goldring MB, Sun HB (2003). CITED2-mediated regulation of MMP-1 and MMP-13 in human chondrocytes under flow shear. *Journal of Biological Chemistry*.

[154] Leong DJ, Li YH, Gu XI (2011). Physiological loading of joints prevents cartilage degradation through CITED2. *FASEB Journal*.

[155] Gerwin N, Hops C, Lucke A (2006). Intraarticular drug delivery in osteoarthritis. *Advanced Drug Delivery Reviews*.

[156] Caron JP, Fernandes JC, Martel-Pelletier J (1996). Chondroprotective effect of intraarticular injections of interleukin-1 receptor antagonist in experimental osteoarthritis: suppression of collagenase-1 expression. *Arthritis and Rheumatism*.

[157] Yorimitsu M, Nishida K, Shimizu A (2008). Intra-articular injection of interleukin-4 decreases nitric oxide production by chondrocytes and ameliorates subsequent destruction of cartilage in instability-induced osteoarthritis in rat knee joints. *Osteoarthritis and Cartilage*.

[158] Jin M, Emkey GR, Siparsky P, Trippel SB, Grodzinsky AJ (2003). Combined effects of dynamic tissue shear deformation and insulin-like growth factor I on chondrocyte biosynthesis in cartilage explants. *Archives of Biochemistry and Biophysics*.

[159] Lima EG, Bian L, Ng KW (2007). The beneficial effect of delayed compressive loading on tissue-engineered cartilage constructs cultured with TGF-*β*3. *Osteoarthritis and Cartilage*.

[160] Helmark IC, Mikkelsen UR, Borglum J (2010). Exercise increases interleukin-10 levels both intraarticularly and peri-synovially in patients with knee osteoarthritis: a randomized controlled trial. *Arthritis Research and Therapy*.

[161] Anghelina M, Sjostrom D, Perera P, Nam J, Knobloch T, Agarwal S (2008). Regulation of biomechanical signals by NF-*κ*B transcription factors in chondrocytes. *Biorheology*.

[162] Perera PM, Wypasek E, Madhavan S (2010). Mechanical signals control Sox-9, Vegf and c-Myc expression and cell proliferation during inflammation via integrin-linked kinase, B-Raf, and ERK 1/2-dependent signaling in articular chondrocytes. *Arthritis Research and Therapy*.

[163] Bao F, Chen Y, Schneider KA, Weaver LC (2008). An integrin inhibiting molecule decreases oxidative damage and improves neurological function after spinal cord injury. *Experimental Neurology*.

[164] Salter DM, Wright MO, Millward-Sadler SJ (2004). NMDA receptor expression and roles in human articular chondrocyte mechanotransduction. *Biorheology*.

[165] Ramage L, Martel MA, Hardingham GE, Salter DM (2008). NMDA receptor expression and activity in osteoarthritic human articular chondrocytes. *Osteoarthritis and Cartilage*.

[166] McGlashan SR, Cluett EC, Jensen CG, Poole CA (2008). Primary Cilia in osteoarthritic chondrocytes: from chondrons to clusters. *Developmental Dynamics*.

[167] Garcia M, Knight MM (2010). Cyclic loading opens hemichannels to release ATP as part of a chondrocyte mechanotransduction pathway. *Journal of Orthopaedic Research*.

[168] Xing Y, Gu Y, Gomes RR, You J (2011). P2Y(2) receptors and GRK2 are involved in oscillatory fluid flow induced ERK1/2 responses in chondrocytes. *Journal of Orthopaedic Research*.

[169] Salter DM, Millward-Sadler SJ, Nuki G, Wright MO (2002). Differential responses of chondrocytes from normal and osteoarthritic human articular cartilage to mechanical stimulation. *Biorheology*.

[170] Millward-Sadler SJ, Wright MO, Lee HS, Caldwell H, Nuki G, Salter DM (2000). Altered electrophysiological responses to mechanical stimulation and abnormal signalling through *α*5*β*1 integrin in chondrocytes from osteoarthritic cartilage. *Osteoarthritis and Cartilage*.

